# 2D KBr/Graphene Heterostructures—Influence on Work Function and Friction

**DOI:** 10.3390/nano12060968

**Published:** 2022-03-15

**Authors:** Zhao Liu, Antoine Hinaut, Stefan Peeters, Sebastian Scherb, Ernst Meyer, Maria Clelia Righi, Thilo Glatzel

**Affiliations:** 1 Department of Physics, University of Basel, 4056 Basel, Switzerland; antoine.hinaut@unibas.ch (A.H.); sebastian.scherb@unibas.ch (S.S.); ernst.meyer@unibas.ch (E.M.); 2 Department of Physics and Astronomy, University of Bologna, 40127 Bologna, Italy; stefan.peeters@unibo.it (S.P.); clelia.righi@unibo.it (M.C.R.)

**Keywords:** AFM, friction, work function, KBr, graphene, Ir(111)

## Abstract

The intercalation of graphene is an effective approach to modify the electronic properties of two-dimensional heterostructures for attractive phenomena and applications. In this work, we characterize the growth and surface properties of ionic KBr layers altered by graphene using ultra-high vacuum atomic force microscopy at room temperature. We observed a strong rippling of the KBr islands on Ir(111), which is induced by a specific layer reconstruction but disappears when graphene is introduced in between. The latter causes a consistent change in both the work function and the frictional forces measured by Kelvin probe force microscopy and frictional force microscopy, respectively. Systematic density functional theory calculations of the different systems show that the change in work function is induced by the formation of a surface dipole moment while the friction force is dominated by adhesion forces.

## 1. Introduction

Heterogeneous materials with different vertically stacking modes have attracted attention for the development of materials with advanced functions [1,2]. On the nanoscale, the family of two-dimensional (2D) materials offers a broad basis for the formation of heterostructures with far-reaching properties and a high application potential [3,4]. Graphene is one of the most outstanding materials due to its properties as a semimetal [5]. In addition, other 2D materials can also be used to establish heterostructures, such as semiconductors (e.g., MoS_2_) and insulators (e.g., h-BN) [6,7,8]. They differ significantly in their behaviors depending on their bulk composition and therefore offer several additional possibilities for observing interesting phenomena [9].

Work function and friction are two fundamental surface properties, but generally they seem to be weakly related. However, for bulk materials, a correlation between the friction coefficient and the work function was observed in 3d transition metals [10,11]. Moreover, on the nanoscale, an increasing work function was measured from a monolayer to a bilayer of graphene on SiC(0001) [12], while the frictional force decreased [13]. These results suggest that both the work function and the friction in homogeneous materials might be related in general [14]. However, the reasons for this are not yet known.

Atomic force microscopy (AFM) is a powerful tool for imaging surfaces at the atomic level in different environments, especially studying the properties of 2D structures. In combination with Kelvin probe force microscopy (KPFM), it offers the possibility to measure contact potential differences (CPD) on heterogeneous surfaces and thus allows obtaining detailed electronic information of the surface [15,16]. Additionally, friction force microscopy (FFM), working in contact AFM mode, is an effective approach to characterize friction processes at the micro- and nanoscale [17,18,19].

Here, the properties of 2D layered heterostructures consisting of a monolayer KBr plus a graphene layer are investigated experimentally and theoretically by evaluating the work function and friction on the nanoscale. Two types of KBr structures were grown, on and next to a layer of graphene. The Ir(111) surface was chosen as the metal substrate because of the formation of a reconstructed KBr structure and easily prepared graphene by decomposing ethene in a chemical vapor deposition (CVD) process. All sample preparations and experiments were carried out in ultra-high vacuum (UHV). Non-contact atomic force microscopy (nc-AFM) was used as the main method to study the sample topography from large scale to atomic resolution, as well as KPFM and FFM to obtain additional surface information. Combined with the simulation results, it shows that the changes in work function and frictional properties are similar for the two KBr structures, which are directly correlated with the formation of a surface dipole and mechanical adhesion forces, respectively.

## 2. Materials and Methods

### 2.1. Sample Preparation

The Ir(111) single crystal sample (MaTeck GmbH, Julich, Germany) was prepared with several cycles of Ar${}^{+}$ sputtering and annealing at 1450 K. To obtain the different coverages of graphene, two different routines were applied in this work. First, the Ir(111) surface was exposed to an ethylene doser for 30 s at a chamber pressure of $2\times 10^{-7}$ mbar to ensure saturation coverage. After annealing to 1350 K for another 60 s, the graphene flakes cover about 20% of the substrate. For the purpose of convenient friction measurement, a second route obtaining a fully covered monolayer graphene was used by the exposure of the hot iridium surface ( 1350 K) to ethylene at the same pressure for 60 s. The KBr powders were evaporated at 700 K to the prepared graphene covered Ir(111) surface at room temperature under UHV conditions via an e-beam evaporator with quartz Knudsen cells.

### 2.2. Atomic Force Microscopy

All the experiments were performed in a home-built UHV RT-AFM microscope operating at a base pressure of $5\times 10^{-11}$ mbar. The images in nc-AFM mode were scanned with stiff silicon cantilevers (PPP-NCL, Nanosensors, k=48 N/$m^{-1}$). Bimodal AFM mode was used to combine first flexural oscillation (resonance frequency of $f_{1}=172$ kHz) with torsional oscillation (resonance frequency of $f_{t}=1.5$ MHz) [20,21]. The applied bias voltage is used to compensate the work function difference between tip and sample [16]. FFM with soft silicon cantilevers (PPP-CONT, Nanosensors, k=0.2 N/$m^{-1}$) was applied to measure the lateral force [22]. Then, the friction force is calculated from the work of enclosed lateral-force hysteresis loop, and averaged by the 256 pairs of lines for every measured area. The adhesion force is measured from the maximum value of the force-distance curve during the force spectroscopy. KPFM was performed in frequency modulation. The frequency of the AC excitation was set to $f_{\mathrm{AC}}=210$ Hz and the amplitude of bias to $U_{\mathrm{AC}}=700$ mV, while the oscillation amplitude of the frequency shift was compensated for by controlling the applied DC voltage [16]. The filtered image in Figure S2 is analyzed via Fast Fourier Transform (FFT) in the software of Gwyddion.

### 2.3. Computational Method

All of the Density Functional Theory (DFT) calculations were performed following the computational approach described in our previous publication [23]. The local density approximation, using the Perdew-Zunger (PZ) [24] parameterization, was chosen to describe the exchange correlation functional. The calculations were performed using periodic supercells and following the pseudopotential/plane-waves computational approach available in the Quantum ESPRESSO computational suite [25,26]. Spin-polarization was taken into account in all the calculations. The plane-wave expansion of the electronic wave function (charge density) was limited to a kinetic energy cutoff of 30 Rydberg ( 240 Ry), since the pseudopotentials used in this work were ultrasoft. We did not take into account dispersion forces, as we did in our previous works [27,28,29,30], because these corrections often overestimate the bond energies involving metallic substrates [31,32]. A Gaussian smearing of 0.002 Ry was used to better describe the electronic occupations around the Fermi level. The cell sizes were $4\times 2\sqrt{3}$ and $5\times 2\sqrt{3}$ for the KBr/Ir(111) and the KBr/Gr/Ir(111) systems, respectively. The former was shown in our previous work to be the ideal size to represent the KBr reconstruction observed experimentally, while the latter offers the best compromise to minimize the lattice mismatch between the three materials. A 5 × 5 × 1 k-point grid was used to sample the Brillouin zone of the systems, based on the 12 × 12 × 12 grid originally selected for the iridium bulk. The convergence threshold for the electronic self-consistent calculations was $1\times 10^{-8}$ Ry, while the geometry optimizations were stopped when the total energy and the forces converged under thresholds of $1\times 10^{-4}$ Ry and $1\times 10^{-3}$ Ry/bohr, respectively. The adhesion energies were calculated as follows:

(1) $$E_{\mathrm{ads}}=E_{\mathrm{tot}}-E_{\mathrm{top}}-E_{\mathrm{bottom}},$$

where $E_{\mathrm{tot}}$, $E_{\mathrm{top}}$ and $E_{\mathrm{bottom}}$ are the total energy of the whole system, the total energy of the adsorbed portion of the system, and the total energy of the substrate, respectively.

For the Bader charge analysis, the code by Henkelman’s group [33,34,35,36] was used, and the details concerning this approach are reported in our recent work [23]. For the calculation of the work functions, symmetric systems were generated to avoid the formation of any electric dipoles in the vacuum region due to the periodic boundary conditions.

The calculation of the charge density difference is similar to the one for the adhesion energy. The charge density of the isolated regions above and below the interface are subtracted from the charge density of the whole system [37].

The XCrySDen software was used to represent the computational systems [38].

## 3. Results

### 3.1. Structural Characterization

**Figure 1:**
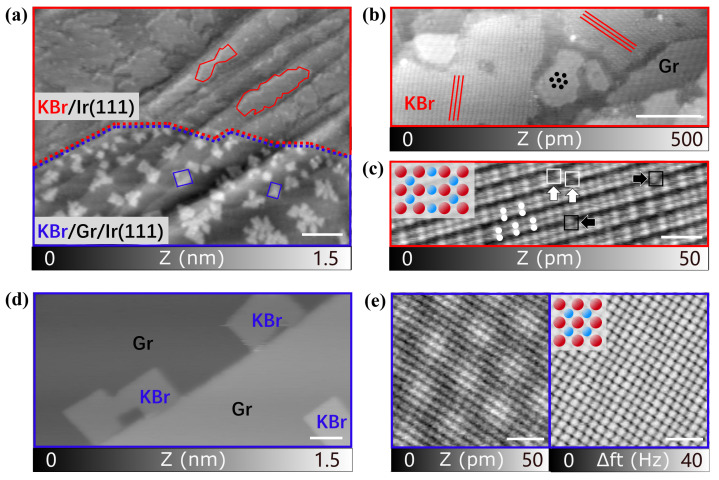
nc-AFM images of KBr accompanied with graphene on an Ir(111) surface: (**a**) Topography with both irregular KBr (top in red) and cubic KBr (bottom in blue). (**b**) Topography of KBr islands rotated in two directions (red lines) and graphene moiré (black spots) on Ir(111). (**c**) Atomic resolution of double parallel lines of reconstructed KBr on Ir(111), with lattice vacancies and adatoms marked with white and black squares, as well as pairs of protrusions marked with white spots, respectively, (the inset shows the atomic configurations with Br in red and K in blue). (**d**) Topography of cubic KBr on Gr/Ir(111). (**e**) Atomic resolution of cubic KBr on graphene on Ir(111), with the left topography image showing the graphene moiré and the right corresponding to the torsional frequency shift illustrating the cubic KBr lattice taken simultaneously at the same region. Scale bars for (**a**): 100 nm, (**b**,**d**): 20 nm and (**c**,**e**): 2 nm.

KBr deposited on an Ir(111) substrate partially covered with graphene is observed both on the raw metal surface and on the 2D graphene layer. As can be seen in Figure 1a, the KBr forms two distinctly different structures. On the upper part of the image, where KBr is directly bound to the metal, there are irregularly shaped monolayers (outlined in red). On the graphene(Gr)/Ir(111) areas in the lower part (outlined in blue) the situation is completely different, with a formation of square shaped islands. KBr shows similar coverage in both regions but a different growth, i.e., larger islands on Ir(111) and smaller islands on Gr/Ir(111). Within the irregularly shaped KBr islands directly on Ir(111), a rippled pattern with tilted stripes can be observed, as shown in Figure 1b. A total of three different stripe directions with 120 ${}^{\circ }$ indicate an alignment of the islands to the substrate surface due to the hexagonal metal lattice symmetry of the (111) surface orientation. Generally, each island has only one type of orientation, and behaves fragile during the scanning (see Figure S1).

At the atomic level, a single stripe in the high-resolution image of Figure 1c consists of two parallel rows with alternating higher and one lower section (highlighted by white dots), with the more obvious image filtered by FFT shown in Figure S2. In addition, several defects can also be observed within or between the lines, in particular pairs of vacancies on top of and free ions between the stripes, represented with white and black squares, respectively. The high density of defects within the KBr islands compared to typical observations of alkali halides on metal surfaces is due to a large lattice strain in this reconstruction as we have shown before [23]. This reconstruction is quite different in comparison to the known cubic KBr reconstruction on most metal surfaces [39,40]. In contrast, when KBr is deposited on Gr/Ir(111), as shown in the lower half of Figure 1a, most of KBr islands are arranged in a more ordered form as detailed in Figure 1d. The high-resolution image in Figure 1e shows the surface details of these sandwich heterostructures on top of a KBr/Gr/Ir(111) island. The overlapping periodicities of the cubic lattice configuration of KBr and the wavy moiré pattern of graphene on Ir(111) are both visible in the topographic nc-AFM measurement in Figure 1e on the left. The image on the right shows the simultaneously measured torsional frequency shift, which is more sensitive to the short-range forces of the cubic KBr lattice. These measurements prove that the KBr layer has grown on top of the graphene layer and that the relatively strong interaction of the graphene with the Ir(111) surfaces leading to the moiré is still intact.

### 3.2. Work Function & Formation of Dipoles

Table 1 shows the experimentally measured and calculated work function of the different systems measured by KPFM [12] and determined by Density Functional Theory (DFT), respectively [23]. If we take the work function measured on Gr/Ir(111) as a reference, the reconstructed KBr directly on Ir(111) shows a −500 meV difference. However, when the cubic KBr is grown on Gr/Ir(111), as shown in Figure 1d, the work function is +150 meV higher than for graphene. These values agree quite well with the results of the DFT calculations. They indicate a pronounced shift in the work function of the KBr monolayer after adding a single layer of graphene.

**Figure 2:**
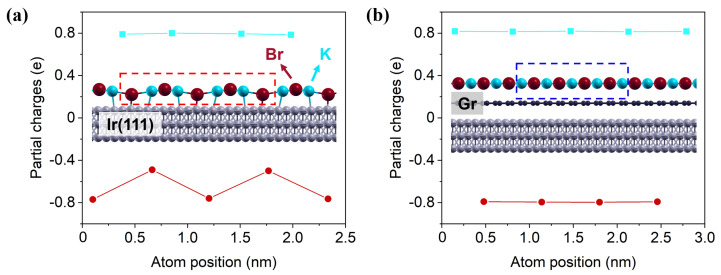
Simulated model (lateral view) of both KBr structures with the partial charges in the dotted box of the model calculated by the Bader method (K in blue, Br in red, C in black and Ir in gray): (**a**) KBr/Ir(111), (**b**) KBr/Gr/Ir(111).

To illustrate the change in work function, the charge distribution of the two different KBr structures was calculated with DFT, as can be seen in Figure 2 showing the side view of the simulated models [23]. For the reconstructed KBr on Ir(111), the anions (Br${}^{-}$) and cations (K${}^{+}$) are shifted into different sublayers, introducing a charge separation and creating a dipole moment perpendicular to the iridium surface ( +0.18 e per KBr unit in total). It is thought to alter the Fermi level [41] and play an important role in the modified and experimentally observed electronic properties. Considering the ability to transport an electron to the outside, the slightly positive KBr layer forms a more favorable channel for an electron escaping into vacuum and results in a lower work function. In contrast, a nearly neutral charge state is calculated for cubic KBr on Gr/Ir(111) ( +0.01 e per KBr unit). The decoupling by adding a single layer of graphene allows the KBr layer to return to electronic equilibrium, leading to an increase in the work function.

### 3.3. Friction and Adhesion Forces

**Figure 3:**
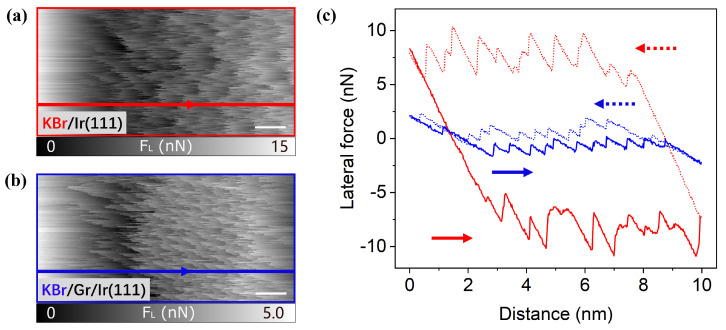
Frictional images (forward direction of the two KBr structures at a normal load of $F_{n}=5$ nN: (**a**) KBr/Ir(111), (**b**) KBr/Gr/Ir(111). Scale bar: 1 nm. The corresponding friction loops of a single line marked in both images are presented in (**c**).

The frictional force on the single-layer KBr islands, measured as the lateral movement of the tip position sliding back and forth, provides additional information about these structures. In Figure 3, measurements of the friction force are shown with a normal load of $F_{n}=5$ nN. The KBr on plain Ir(111) shows a friction force of 6.02±0.38 nN, calculated from the area enclosed by the frictional hysteresis loop. This is quite a large value compared to bulk KBr, where the friction force is usually below 1 nN at the same load both in experiments [42] and calculations [43]. With an intercalated graphene layer the friction force of KBr on top decreases to 0.48±0.04 nN to a similar order of magnitude as for bulk KBr. The question arises as to how the large discrepancy between the friction of the two KBr types can be explained. Therefore, the adhesion force was also measured and compared to the former results to investigate whether the effect is electronically or mechanically dominated.

**Figure 4:**
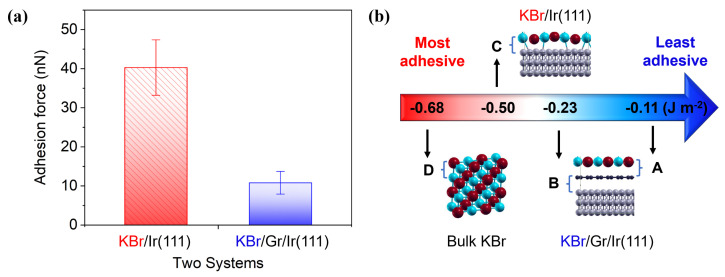
Adhesion force and energy of KBr on Ir(111) and Gr/Ir(111) evaluated by: (**a**) force spectroscopy in FFM and (**b**) DFT simulations.

The adhesion force was measured on the two KBr surfaces by force spectroscopy. As summarized in Figure 4a, the adhesion forces of the reconstructed KBr layer on Ir(111) are almost four times larger than those of cubic KBr on Gr/Ir(111). This indicates that the strong interaction with the iridium substrate has an influence on the tip contact forces through the reconstructed KBr layer. This effect is mitigated by the middle graphene layer with the corrugated superstructures, resulting in a lower friction force on cubic KBr for the sliding tip. This suggests that the differences between the adhesion forces for the two KBr types are due to the effect of the decoupling graphene rather than the substrate.

To verify our measurements, the adhesion energy at the selected interfaces is calculated using DFT. Unsurprisingly, the energy between the reconstructed KBr and the iridium substrate (interface C) in Figure 4b is the most negative and even approaches the ionic bond between the KBr interlayers (interface D). In comparison, the energy at interface B between graphene and Ir(111) decreases to only half, due to the decoupling effect and the large interstitial space (almost 0.34 nm [44]). Because of this interfacial shielding, the lowest adhesion energy is observed between the cubic KBr and the graphene (interface A), with only 22% of interface C, which fits well with the four times larger adhesion force of the reconstructed KBr compared to the cubic KBr. The lower binding energy was also confirmed by the successful manipulation of a rectangular KBr island (see Figure S3a). As can be seen in Figure S3b, the KBr island was altered by the scanning tip, here with the scan direction pointing upwards. This indicates that KBr resting on graphene can be easily moved even with a non-contact tip. Comparing to the physisorbed graphene on Ir(111) (interface B) [45], the only half adhesion energy between KBr and graphene (interface A) indicates a quite weak Van der Waals connection, thus leading to an easier relative sliding in Figure S3.

**Figure 5:**
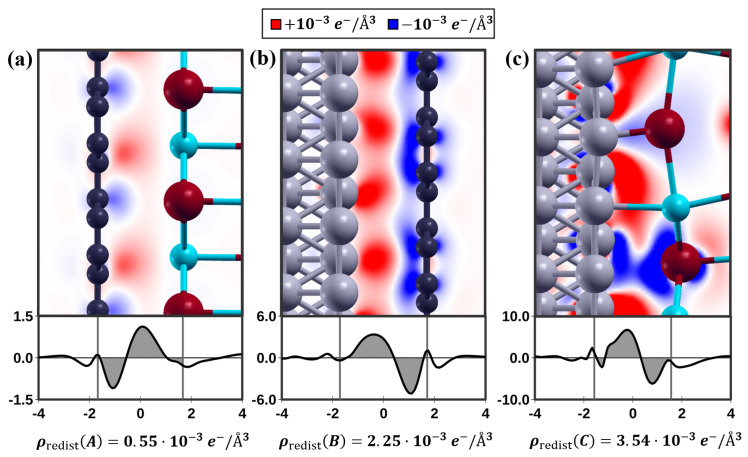
Top: charge density differences ρ${}_{\mathrm{diff}}$ of (**a**) KBr on Gr, (**b**) Gr on Ir(111) and (**c**) KBr on Ir(111). Red and blue regions represent charge accumulation and depletion, respectively. Bottom: $\bar{\rho }$${}_{\mathrm{diff}}\left(z\right)$, i.e., planar averages of ρ${}_{\mathrm{diff}}$. The horizontal axis indicates the height in angstrom and is centered on the middle of the interface. The vertical axis indicates the values $\bar{\rho }$${}_{\mathrm{diff}}\left(z\right)$ multiplied by $10^{3}$. The amount of redistributed charge, ρ${}_{\mathrm{redist}}$, is indicated by the gray shaded areas on the graph below.

The corrugated K${}^{+}$ and Br${}^{-}$ ions cause a local redistribution of charges on the Ir(111) surface, as shown in Figure 5. The calculation of the charge density difference ρ ${}_{\mathrm{diff}}\left(r\right)$ is useful to visualize the redistribution of the electrons arising when two isolated systems are brought into contact. ρ ${}_{\mathrm{diff}}\left(r\right)$ is calculated as the difference between the electronic charge density of the two interacting systems ρ ${}_{1+2}\left(r\right)$ and the sum of the charge densities of the two isolated systems ρ ${}_{1}\left(r\right)$ and ρ ${}_{2}\left(r\right)$:

(2) $$\rho_{\mathrm{diff}}\left(r\right)=\rho_{1+2}\left(r\right)-\rho_{1}\left(r\right)-\rho_{2}\left(r\right).$$

Red and blue regions in Figure 5 correspond to charge accumulation and depletion, respectively. A figure of merit useful to quantify the amount of charge that is redistributed at the contact is ρ ${}_{\mathrm{redist}}$ [37], which can be obtained as follows:

(3) $$\rho_{\mathrm{redist}}=\frac{1}{2z_{0}}\int_{-z_{0}}^{z_{0}}\left|{\bar{\rho }}_{\mathrm{diff}}\left(z\right)\right|\mathrm{dz},$$

where $\bar{\rho }$ ${}_{\mathrm{diff}}\left(z\right)$ represents the planar average of ρ ${}_{\mathrm{diff}}\left(r\right)$ and $z_{0}$ is half of the interfacial distance. Interface A, characterized by the weakest adhesion, showcases the lowest charge redistribution, while interface C has the highest charge redistribution and the strongest adhesion. The connection between interfacial adhesion and charge redistribution was already shown in a previous work [37] and is confirmed to be valid for these systems as well. The formation of the reconstruction alters the electronic properties of KBr and lowers the work function of the system. However, the strong adhesion forces that are observed increase the interaction between the silicon tip of the microscope and the reconstructed KBr surface. This allows for additional dissipation of the tip’s kinetic energy, which corresponds to higher friction. Thanks to the graphene intercalation, the top KBr layer returns to an incommensurable state with a cubic configuration, whose adhesion force with the sliding tip is expected to be weaker compared to its reconstructed phase. Therefore, the energy is almost conserved by converting the kinetic energy into electrodynamic potential energy and returning to the original form due to the lower frictional force [46]. In summary, all results indicate that the frictional forces of such heterostructures on the nanoscale are rather determined by the adhesion forces, while the displacement of the ions by the interaction with the substrate induces a surface dipole and thus directly influence the work function.

## 4. Conclusions

We prepared single layer KBr on Ir(111): (i) a corrugated layer directly on the substrate with a strong adhesion and (ii) a flat one with intercalated graphene resulting in a weaker adhesion. For both, we determined, in addition to the structure and adhesion, the work function and friction properties. In nc-AFM experiments, a KBr reconstruction with a stripe-like periodicity was observed directly on Ir(111). When growing KBr on a monolayer of graphene on the same substrate, the structure reverts to the cubic lattice configuration. The frictional properties measured by FFM show that the reconstructed KBr has a significantly larger friction force than the cubic KBr, due to larger interfacial adhesion. DFT calculations demonstrated the connection between adhesion and the charge redistributed at the different interfaces. Larger adhesion forces lead to a considerable energy dissipation in the frictional hysteresis loop. Furthermore, the work function measured by KPFM and calculated by DFT increases from reconstructed to cubic KBr, which we traced back to the formation of an electrical dipole moment in the KBr layer. This sheds light on the dominant factors behind the work function and frictional properties of 2D materials, and helps to control and modify them through different compositions of the heterostructures.

## Figures and Tables

**Table 1:** Work function values experimentally observed by KPFM measurements and theoretically by DFT simulation results [23].

System	$\Phi_{\mathrm{EXP}}$ (eV)	$\Phi_{\mathrm{DFT}}$ (eV)
Ir(111)	5.76	5.77
KBr /Gr/Ir(111)	4.71	4.96
Gr/Ir(111)	4.56	4.74
KBr/Ir(111)	4.06	4.16

## Data Availability

Not applicable.

## Supplementary Materials

The following are available online at https://www.mdpi.com/article/10.3390/nano12060968/s1, Figure S1: nc-AFM images of a KBr island on Ir(111) before and after with a missing arm. Figure S2: Atomic reconstructed KBr lattice on Ir(111) measured by nc-AFM and filtered by FFT, respectively. Figure S3: nc-AFM images of a KBr island on Gr/Ir(111) before and after tip manipulation with downward and upward scanning directions, respectively.
