# Supplementary material for: 2D KBr/Graphene Heterostructures—Influence on Work Function and Friction
Source: Nanomaterials (Basel). 2022 Mar 15;12(6):968. doi: 10.3390/nano12060968 (PMC8949013; doi:10.3390/nano12060968)
Supplement: Supplementary file 1 [file nanomaterials-12-00968-s001.zip › nanomaterials-1633242-supplementary.pdf]

Article

Supplementary Materials for: 2D KBr/Graphene Heterostructures – Influence on Work Function and Friction

Zhao Liu [1]*, Antoine Hinaut [1], Stefan Peeters [2], Sebastian Scherb [1], Ernst Meyer [1], Maria Clelia Righi [2] and Thilo Glatzel [1]*

[1] Department of Physics, University of Basel, 4056 Basel, Switzerland
[2] Department of Physics and Astronomy, University of Bologna, 40127 Bologna, Italy
* Correspondence: thilo.glatzel@unibas.ch(T.G.); zhao.liu@unibas.ch (Z.L.)

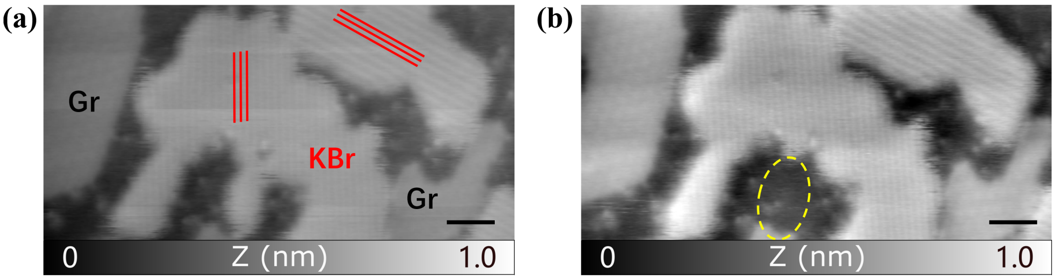

Figure S1. nc-AFM images of a KBr island on Ir(111) before (a) and after (b) with a missing arm (marked in a yellow dashed circle). Scale bar: 10 nm.

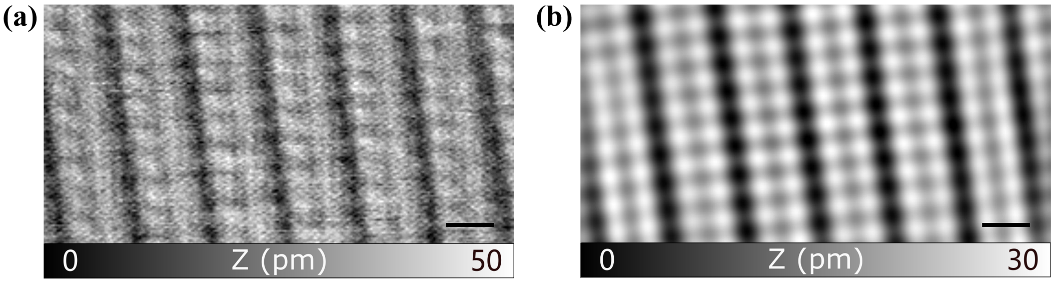

Figure S2. Atomic reconstructed KBr lattice on Ir(111): (a) Measured by nc-AFM. (b) Filtered by FFT. Scale bar: 1 nm.

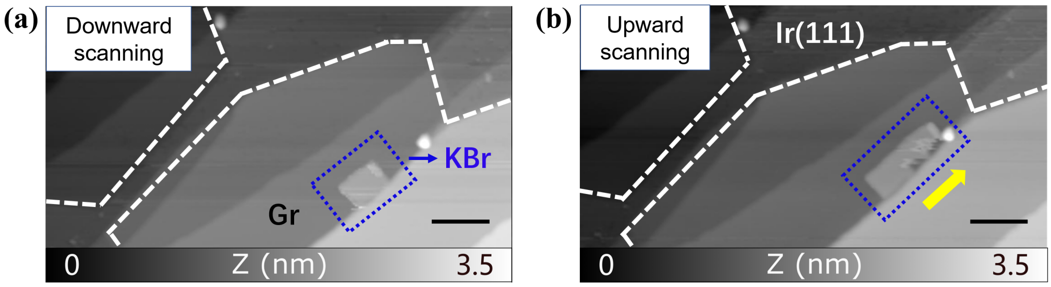

Figure S3. nc-AFM images of a KBr island on Gr/Ir(111) before (a) and after (b) tip manipulation with downward and upward scanning directions, respectively. Scale bar: 30 nm.
